# Impact of Medical School on the Relationship between Nutritional Knowledge and Sleep Quality—A Longitudinal Study of Students at Wroclaw Medical University in Poland

**DOI:** 10.3390/nu16020278

**Published:** 2024-01-17

**Authors:** Aureliusz Andrzej Kosendiak, Bartosz Bogusz Adamczak, Zofia Kuźnik, Szymon Makles

**Affiliations:** 1 Department of Physical Education and Sport, Wroclaw Medical University, 51-601 Wroclaw, Poland; 2 Student Scientific Association, Department of Physical Education and Sport, Wroclaw Medical University, 51-601 Wroclaw, Poland

**Keywords:** students, students’ knowledge, medical university education, diet, feeding behavior, sleep quality, sleep duration, sleep aids, attitude to health, young adult

## Abstract

The aim of this study was to investigate the impact of the first year of medical school on the relationship between nutritional knowledge and sleep. To achieve this, first-year medical students at Wroclaw Medical University were invited to participate in the study during both the initial and final months of their first academic year. The study included 570 students in the initial period and 705 in the latter. The research questionnaire comprised the KomPAN, assessing nutritional knowledge, and the Pittsburgh Sleep Quality Index (PSQI), evaluating sleep quality. The majority of students demonstrated at least sufficient nutritional knowledge, while approximately two-thirds of students experienced poor sleep in both periods. Notably, sleep quality further deteriorated in the second period (PSQI total score: 6.86 vs. 7.38, *p* = 0.0157). This change was influenced mainly by a decrease in sleep duration and an increase in the use of sleep medications (both *p* < 0.0001). The significant difference in overall sleep quality between different nutritional knowledge levels emerged only in the second semester, where students with the highest nutritional knowledge slept the best, while those with the lowest slept the worst (*p* = 0.0001). Crucially, in both periods, the use of sleep medications was highest among individuals with insufficient nutritional knowledge. Throughout the academic year, the usage increased for all except those with the highest nutritional knowledge, who exhibited the best sleep (*p* < 0.0001). The escalating use of sleep medications among medical students warrants greater attention, and leveraging the relationship between nutritional knowledge and sleep could prove beneficial, as positive habits in one domain may positively influence the other.

## 1. Introduction

The inaugural year of medical studies at a medical university represents a pivotal juncture for students as they navigate the complexities of both academic and personal adjustments [1]. This period is marked by a multifaceted array of challenges that extend beyond the traditional boundaries of scholarly pursuits [2]. As students transition from the familiarity of home to the independence of university life, a host of responsibilities associated with solo living come to the fore [3]. The process of transition entails learners autonomously crafting a new identity for themselves as students in higher education [4]. The newfound autonomy involves not only academic endeavors but also essential aspects of daily life, such as independent cooking [5], grocery shopping [6], and a potential restructuring of financial arrangements [7].

Simultaneously, the academic demands of the first year of medical school are inherently demanding, characterized by challenging coursework, an intensive study regimen, and an academic calendar that may deviate from conventional schedules [8]. A study by Shah et al. showed that the most common sources of stress among medical students were related to academic and psychosocial concerns. ‘High parental expectations’, the ‘frequency of examinations’, the ‘vastness of academic curriculum’, ‘sleeping difficulties’, ‘worrying about the future’, ‘loneliness’, ‘becoming a doctor’, and ‘performance in periodic examinations’ emerged as the most frequently and severely encountered stressors [9]. Moreover, research indicates that first-year students report significantly lower levels of social support [10]. These factors collectively influence the daily routines of students, impacting both dietary habits and sleep patterns [11]. The need to strike a delicate balance between academic commitments and personal well-being is further complicated by the introduction of an adult social environment. This phase often signifies an increase in social engagements, fostering new relationships, and adapting pre-existing connections with their parents and families [12].

Within this period of transition, the role of maintaining healthy dietary habits and nutrition assumes heightened significance. Our study aimed to assess the extent of knowledge regarding accurate nutritional principles and its potential impact on the dietary practices and overall nutritional well-being of students [13]. The adoption of sound nutritional practices is crucial, as it significantly contributes to the overall well-being [14] and academic performance of students [15]. To illustrate, a well-balanced diet furnishes the consumer with essential nutrients necessary for cognitive function and sustained concentration during rigorous study sessions [16]. Furthermore, the cultivation of healthy eating habits supports the physical and mental well-being essential for navigating the challenges inherent in the demanding academic environment [17]. The positive impact of nutrition extends beyond the academic realm, influencing aspects such as stress management, energy levels, and overall resilience [18]. Given these considerations, fostering a commitment to a wholesome diet emerges as a cornerstone in promoting the holistic health and success of students in their inaugural year of medical studies.

In the context of students’ well-being during this transitional phase, the significance of maintaining a healthy sleeping routine cannot be overstated. A robust sleep regimen is a vital component of overall health, influencing the immune system and immunological response [19]. Sleep loss, inadequate sleep duration, and other sleep disturbances have been linked to heightened inflammation, impairing adaptive immunity [20]. The short-term consequences of sleep disruption encompass somatic pain, emotional distress, mood disorders, and memory deficits, while long-term repercussions may include hypertension, cardiovascular disease, and weight-related disorders [21]. Notably, researchers have extensively explored the sleep hygiene of medical students. Their findings reveal an increased prevalence of sleep disturbances and a lower quality of sleep when compared to diverse control groups [8,22]. The matter is of heightened concern, as sleep disorders have been shown to exhibit a correlation with burnout among medical students [23]. Recognizing the profound impact of sleep on various aspects of health, including cognitive function, emotional well-being, and physical health, underscores the importance of fostering healthy sleep habits as an integral component in supporting the comprehensive well-being and success of students during their inaugural year of medical studies.

Within this evolving landscape, the amalgamation of academic rigor and the social dimensions of early adulthood may contribute to heightened stress levels, exhaustion [10], disruptions in sleep patterns [8], and modifications in dietary practices [5]. Rather than delving into the extensively researched field of the relationship between stress levels and sleep quality [24,25,26,27,28], we aimed to contribute insights into the less explored relationship between nutritional knowledge and sleep quality, providing a nuanced perspective on the well-being of these students.

The interplay between health-related habits is a subject of considerable interest in the field of public health and medical research. Numerous studies have suggested a reciprocal relationship among various health-related behaviors, where the adoption of one positive habit may catalyze the improvement of others [29,30,31,32]. Investigating these relationships is crucial for developing comprehensive strategies to enhance overall well-being. One such aspect under scrutiny is the relationship between dietary habits and sleep quality [33]. Recognizing that health-related behaviors often intersect, our study focuses on the examination of nutritional knowledge as a potential influencer on various health habits, particularly its impact on the quality of sleep among medical students. This exploration is rooted in the hypothesis that a well-informed nutritional approach may extend its positive influence beyond dietary choices, potentially affecting sleep patterns and overall sleep quality. By delving into these connections, we aim to contribute to a nuanced understanding of the intricate dynamics among health-related behaviors, paving the way for more targeted and holistic health interventions.

The objective of the present study is to investigate the quality levels of various sleep domains relative to the level of nutritional knowledge, and how these relationships evolved among students enrolled at Wroclaw Medical University during the course of their inaugural year of studies, a period recognized for its inherent stressors. The examination of these particular factors assumes paramount importance within this specific cohort, given their future roles in medical professions, necessitating a heightened resilience to stressors [34,35]. Moreover, the expectation for these individuals to advocate for healthier lifestyles among their patients, coupled with the integral social role they perform [36,37], underscores the significance of scrutinizing their own habits and coping mechanisms. Thus, it becomes imperative to monitor the varied habits and coping strategies employed by students pursuing degrees in medical-related disciplines.

## 2. Materials and Methods

### 2.1. Study Design and Participants

The study was meticulously planned as a longitudinal examination involving first-year students at Wroclaw Medical University. This design was chosen to facilitate a robust comparison of observed changes over time. The overarching aim of the study was to investigate the impact of medical studies on the relationship between nutritional knowledge and sleep patterns.

To delve into this dynamic, data collection occurred during two distinct periods: the initial month of the academic year (AY) in October 2022 and the concluding month of the first year in June 2023. During these phases, students who engaged in mandatory physical education (PE) classes were provided with links to fully anonymous surveys.

To ensure the broadest representation, invitations were extended to all students who potentially met the inclusion criteria. This inclusivity aimed at capturing diverse perspectives within the cohort.

A paramount aspect of the study design was the meticulous consideration of ethical principles. All participants were informed of the study’s purpose, and their voluntary participation was sought through a process of informed consent.

**Figure 1:**
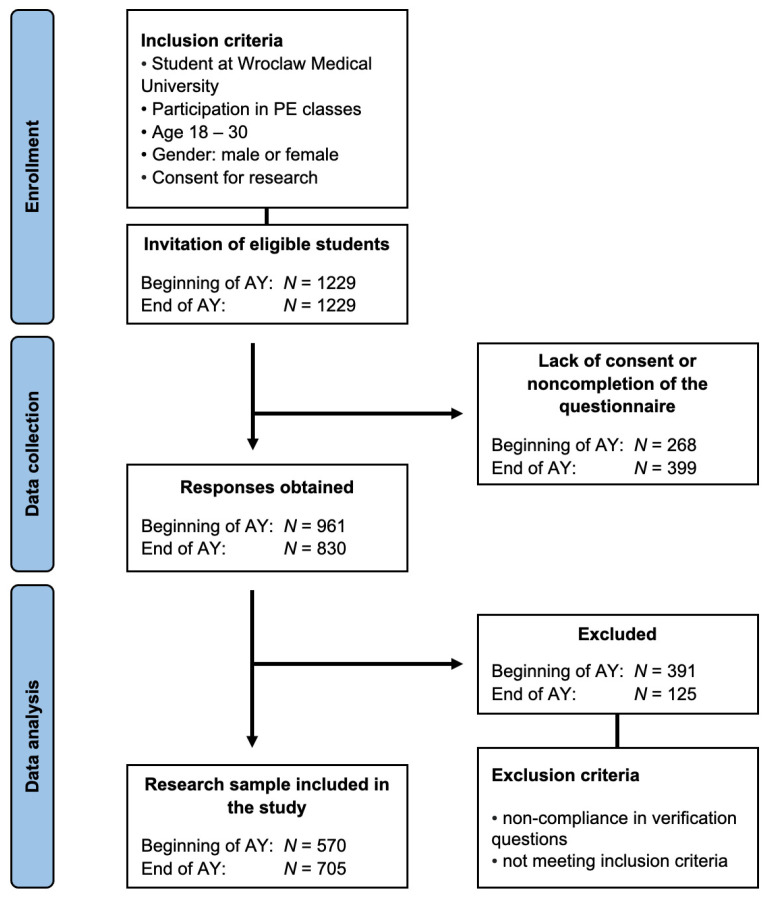
Study selection process.

For a detailed overview of the data collection process and inclusion and exclusion criteria, readers are referred to Figure 1.

### 2.2. Selection of Study Variables

Acknowledging the recognized inverse correlation between questionnaire length, result reliability, and response rate [38,39], our study design deliberately adopts a concise format, focusing exclusively on two aforementioned variables—nutritional knowledge and sleep quality. This decision not only fortifies internal validity but also enhances the interpretability of findings, potentially augmenting the utility of our research. 

The choice of sleep quality is backed by its easy measurability and a role as a key predictor of individual well-being and lifestyle choices [40]. The selection of nutritional knowledge is grounded in its inherent adaptability to modification through diverse lifestyle interventions. This adaptability is particularly noteworthy in the context of social interventions and educational initiatives, especially among medical students, who inherently engage with dietary knowledge in their curriculum [41,42]. Other variables, such as stress or social interactions, may also exert an influence on sleep quality; however, given the challenges associated with modifying them, any identified relationships may offer limited utility in the context of social interventions.

### 2.3. KomPAN

The KomPAN [43] is a comprehensive survey tool developed by the Polish Academy of Sciences. It is used as a robust instrument for the evaluation of dietary habits and nutrition beliefs in individuals.

A key segment of the questionnaire entails a single-choice nutrition beliefs test, encompassing 25 questions meticulously designed to probe the respondent’s comprehension of food and nutrition. Respondents are presented with True/False/I’m not sure answer options, with one point allocated for a correct response, while zero points are awarded for an incorrect or uncertain reply. The resultant scores categorize the respondent’s knowledge into three tiers: insufficient (0–8 points; 0–32%), sufficient (9–16 points; 36–64%), or good (17–25 points; 68–100%).

To fortify the questionnaire’s integrity, three sets of validation questions are strategically embedded to authenticate respondent reliability and facilitate the exclusion of untrustworthy responses from subsequent analyses.

It is noteworthy that the KomPAN questionnaire on nutrition has undergone a rigorous validation process, emerging as a dependable and valuable tool for researchers delving into the intricate realms of dietary habits and nutrition beliefs among individuals [44].

### 2.4. The Pittsburgh Sleep Quality Index (PSQI)

The PSQI [45] is a comprehensive questionnaire comprising 19 self-rated inquiries that pertain to the preceding 4-week period. This instrument meticulously assesses seven distinct components pivotal to gauging the quality of sleep. Additionally, it includes five questions intended for evaluation by a cohabitant, although these were excluded from our analysis. This exclusion stems from their non-inclusion in the overall scoring methodology, coupled with the prevalence of solo sleeping among the majority of our student participants.

Each component is assigned a score ranging from zero (indicating the absence of any issue) to three (reflecting the most severe problem). The cumulative scores across these components contribute to the determination of the total score, which spans from 0 (indicating optimal sleep quality) to 21 (indicating the most suboptimal sleep quality). For our analysis, a threshold of more than five points in the Total Score was considered indicative of poor sleep quality.

The specific components assessed by the PSQI include:

- Sleep duration (DURAT): Awarding zero points for achieving 7 or more hours of sleep.
- Sleep disturbances (DISTB): A scoring system dependent on the presence of disturbances affecting the continuity of night sleep, such as feeling too hot or too cold, experiencing unsettling dreams, or discomfort in breathing.
- Sleep latency (LATEN): Allocating zero points for a latency period of less than 15 min and three points for a latency exceeding 60 min.
- Daytime dysfunctions (DAYDYS): Scoring based on the frequency with which a lack of nocturnal rest impacts daytime behavioral disruptions, such as eating habits or participation in meetings.
- Habitual sleep efficiency (HSE): Scoring contingent on the ratio of actual hours of sleep to the time spent in bed.
- Subjective sleep quality (SLPQUAL): Assigning a score based on the subjective evaluation of sleep quality by the respondent.
- Use of sleep medication (MEDS): Scoring in relation to the frequency of sleep medication usage by the respondent.

This methodology affords a nuanced understanding of the multifaceted dimensions of sleep quality, capturing both subjective and objective aspects for a comprehensive evaluation. 

The PSQI is a questionnaire with established validity and reliability, demonstrated through research, including studies conducted within the Polish population [46]. 

### 2.5. Statistical Analysis

In this study, Microsoft Excel version 16.77 (Redmond, WA, USA) was utilized for the cleansing, curation, and computation of raw data. Statistical analysis, including the identification of significant relationships, was performed using Statistica 13 (Statsoft, Kraków, Poland). 

The data collected in the survey exhibited a non-normally distributed pattern, as determined by the Shapiro–Wilk test. A descriptive analysis was employed, encompassing frequency and percentage measures, for qualitative variables and the mean, median, and interquartile range (IQR) were collected for PSQI outcomes. For the comparison of three or more quantitative variables, the Kruskal–Wallis test was employed, and for the comparison of two quantitative variables, the Mann–Whitney U test was used. Due to the complete anonymization of data collection, conducting a dependent sample statistical test was unfeasible. All analyses were performed at a significance level of *p* < 0.05.

## 3. Results

Table 1 presents the general characteristics of the study participants. The average age of students in both periods was approximately 20 years. Similarly, at the beginning and end of the academic year, around three-quarters of the participants were female. Participants primarily resided in large cities. In both periods, individuals classified as underweight constituted 10–15% of the population, as did those classified as overweight. The majority of participants in both periods had sufficient nutritional knowledge, with individuals rated as good comprising slightly more than a quarter of the participants. However, there was a noticeable almost twofold increase in individuals with insufficient knowledge during the academic year. The quality of sleep was similar in both study periods, with approximately 35% of participants’ responses indicating poor sleep.

Table 2 presents the overall results of sleep quality components and their comparison between the beginning and end of the academic year. It is noteworthy that the average total sleep quality, both at the beginning and end of the academic year, can be characterized as poor; however, it significantly worsened in the later period (*p* = 0.0157, Z = 2.424). This change is attributed to a deterioration in sleep duration (*p* < 0.0001, Z = 7.169) and the increased need for sleep medication (*p* < 0.0001, Z = 5.84). However, there was an improvement in sleep latency over the academic year (*p* = 0.0063, Z = 2.786).

Table 3 presents the relationship between the results obtained in the nutrition beliefs questionnaire, categorized by levels of knowledge, and specific components of sleep quality. It is noteworthy that, at the beginning of the academic year, there was no significant correlation between nutritional knowledge and total sleep quality, represented by total score. However, this correlation emerged in the second semester, where a higher level of nutritional knowledge was associated with better total sleep quality (*p* = 0.0001, H = 19.732). This change could be attributed to a decline in total sleep quality among individuals with nutritional knowledge categorized as sufficient over the academic year, and therefore a better stratification of outcomes (*p* = 0.0127, Z = −2.491).

In the analysis of sleep quality components, it is observed that the duration of sleep significantly deteriorated over the study period in the sufficient and good knowledge groups, while the decline in the insufficient group was statistically insignificant. Regarding sleep disturbance, there were significant differences based on nutritional knowledge in both periods, with the disparity between these groups significantly widening at the end of the academic year. Individuals with insufficient knowledge were particularly susceptible to disruptions in sleep continuity. Sleep latency was not influenced by nutritional knowledge in either period, but individuals with a good rating exhibited a significant improvement in this indicator over the academic year (*p* = 0.0015, Z = 3.167). Daytime dysfunctions due to sleepiness and subjective sleep quality were independent of nutritional knowledge in both periods and remained unchanged across all groups over the study period. Sleep efficiency was unaffected by nutritional knowledge at the beginning of the academic year; however, this difference became apparent during the course of the study. Individuals with insufficient knowledge had significantly lower sleep efficiency than those with a good rating at the end of academic year. Furthermore, individuals with good nutritional knowledge showed a significant improvement in sleep efficiency over the academic year, potentially attributable to a noteworthy enhancement in sleep latency within this group.

Regarding the use of sleep medication, individuals with insufficient nutritional knowledge exhibited a higher frequency of such needs compared to those with sufficient knowledge over the academic year. By the end of the academic year, individuals with an insufficient rating similarly represented a group with an increased need for sleep medication. Furthermore, across all groups except those with a good rating, there was an augmented demand for such measures.

## 4. Discussion

### 4.1. Anthropometric Data

This study sought to assess the correlation between the nutritional knowledge and sleep quality of students enrolled at Wroclaw Medical University at the onset and conclusion of their inaugural year of studies. Wroclaw, an urban center with a resident population exceeding 100,000, accommodates students, some of whom commute from rural areas. The research cohort predominantly comprised women, mirroring the higher representation of female students in medical universities [47].

Regarding the analysis of Body Mass Index (BMI), during both temporal periods, the prevalence of participants categorized as overweight (BMI < 25) or underweight (BMI > 18.5) ranged between 10 and 15%, with the rate of abnormal BMI constituting approximately one-quarter of the respondents. Similar results were obtained in a longitudinal study on Polish nursing students between 2019 and 2021 (23.44% of abnormal BMI on average) [48]. The outcome of our study demonstrates a favorable result in comparison to various studies conducted across diverse populations, such as in Poland (36.3%) [49] and Spain (30.3%) [50]. Conversely, an earlier study in 2022, involving a similar, smaller sample of medical students from Wroclaw, revealed an abnormal BMI rate of 17.5%, representing a notably better outcome [51]. It is pertinent to note, however, that BMI, while widely utilized, may not consistently provide an accurate assessment of excessive body fat. This limitation arises from its inability to differentiate between fat and fat-free mass, including muscles and bones [52]. Consequently, certain students may have been misclassified as overweight when, in actuality, they possessed an athletic build with an appropriate weight.

### 4.2. Sleep Quality

The aim of this study was to evaluate the sleep quality of students at the beginning and end of the academic year. A consistent and healthy sleep routine is considered essential for overall well-being, as disturbances in sleep patterns have been associated with various issues such as inflammation, memory problems, and somatic pain [19]. In the long term, these disturbances may contribute to an increased risk of cardiovascular diseases [20]. Participants in the study were asked to rate their sleep quality in different categories, with higher scores indicating poorer sleep quality. 

It is pertinent to observe that the mean total sleep quality, at both periods of the academic year, can be described as suboptimal, with 62.5% of respondents at the beginning and 65.4% at the end of the academic year having a poor quality of sleep; additionally, there was a notable decline in overall sleep quality, shortened sleep duration, and an increased prevalence of sleep medication utilization towards the end of the academic year. Nonetheless, the seemingly contradictory improved outcomes in sleep latency observed at the end of the academic year may also be associated with a reduction in the available time for sleep due to heightened academic demands. The increased fatigue resulting from the substantial volume of study hours may exert additional pressure on students to expedite the process of falling asleep in an effort to maximize the duration of sleep within the constrained time frame. The results were similar to an Indian study, which found that 64.38% of first-year students were found to have poor sleep quality [53].

Additionally, a 2020 meta-analysis examining sleep quality among medical students revealed a consolidated prevalence of 52.7% for poor sleep quality. Across continents, suboptimal sleep quality exhibited the highest occurrence in Europe, succeeded by the Americas, Africa, Asia, and Oceania [54]. Furthermore, a distinct study indicated that statistically significant likelihoods of “very bad” subjective sleep quality were exclusively observed among students in their initial academic years [55]. These findings align with similar studies, such as that conducted among Greek medical students, where more than half of the participants (52.4%) perceived their sleep quality as poor [56], as well as a Polish study on medical university students, with 52% of them having a poor sleep quality in 2021 [57]. Moreover, a multinational study encompassing medical students reported poor sleep quality in 73.5% of participants, representing an even more alarming prevalence [58]. A parallel trend emerged in a substantial study involving middle-aged healthcare workers, where the average total PSQI score, while still concerning, was lower than that observed among students in our study, suggesting sleep disturbances among medical professionals as well (6.64 compared to 7.38) [59]. Furthermore, an investigation within an elderly adult population yielded a total PSQI score indicative of poor sleep quality (5.69), yet comparatively better than the scores observed in our study [60]. However, the outcomes of our investigation exhibit a notably inferior profile when compared with the findings of the study conducted in Mexico, where only 24% of first-year medical students disclosed encountering sleep-related challenges within the week preceding the survey [61]. 

A noteworthy comparability to the findings in our study was revealed in a 2022 meta-analysis on a population of cancer patients [62]. The total sleep quality scores reported were 7.11 before the initiation of treatment, 8.31 during treatment, and 7.10 after the completion of treatment. Additionally, another meta-analysis focusing on sleep quality among nursing staff demonstrated results akin to ours, indicating a total PSQI score of 7.13 [63]. These congruent findings across diverse populations underline the universality of sleep-related challenges and emphasize the relevance of addressing sleep health across various healthcare settings. However, research on the general population indicates significantly more favorable outcomes. A 2017 German study reported a mean PSQI score of 5.0 for all age groups, with only 36% of individuals perceiving their sleep as of poor quality [64]. Similarly, a general population-based study conducted in China in 2017 revealed poor sleep quality in only 26.6% of participants [65]. Additionally, multiple logistic regression analysis identified factors such as female gender, older age, higher education levels, unmarried status, rural residence, cigarette smoking, and alcohol consumption as associated with insomnia. These findings underscore the concerning disparity between our study’s outcomes and the comparatively better sleep quality observed in the general population, highlighting the need for targeted interventions to address sleep health issues among medical students.

The observed deterioration in sleep quality, shortened sleep duration, and increased utilization of sleep medication among our cohort of medical university students towards the end of the academic year is a concerning trend that warrants careful consideration. Several potential factors may contribute to these adverse changes. The rigorous academic demands, including a high volume of coursework and examinations, may adversely impact sleep patterns [66]. Studies have shown that anxiety and stress may contribute to the exacerbation of overall sleep quality, shortened sleep duration, and increased reliance on sleeping medication [67]. Lifestyle factors, such as heightened caffeine intake [68] or increased screen time [69], may also play a role in compromising sleep hygiene. With medical students exhibiting an elevated consumption of caffeine [70] and increased screen time [71] during examination periods, this can be another factor contributing to their poorer sleep quality at the end of the first year of medical education.

### 4.3. Nutritional Knowledge

Our study discerned that more than 80% of the participants exhibited sufficient or good nutritional knowledge. As nutritional knowledge is recognized to impact dietary habits [72,73], these habits of medical students play a pivotal role in their overall well-being and academic performance [74]. Proper nutrition is integral to cognitive function [75] and sustained attention [76], both of which are imperative for the rigorous learning demands inherent in medical curricula. Furthermore, the prevalence of stress among medical students is well-documented [77,78], and dietary choices can significantly impact stress levels [79,80]. A well-balanced diet, rich in essential nutrients, has been associated with stress resilience [81] and the mitigation of adverse psychological effects [82]. Given the multifaceted challenges encountered during medical training, including long hours of study and the transitional difficulties of first-year students, the role of nutrition in bolstering physical and mental resilience becomes paramount. As such, fostering and promoting good dietary habits among medical students is not merely a matter of personal health but an essential component of optimizing their capacity to excel academically and navigate the stresses inherent in their educational journey [83,84]. 

In a 2019 study involving medical-related students in Poland, comparable findings were observed, indicating a marginally lower level of nutritional knowledge, with an insufficient level of 18.7%, sufficient at 68.2%, and good at 13.1% [85]. A subsequent Polish study conducted in October 2020 amid the COVID-19 pandemic reported even more unfavorable outcomes, revealing an insufficient nutritional knowledge level of 21%, sufficient at 61.5%, and good at 17.5% [86]. Furthermore, an investigation focusing on first-year physical education students in Warsaw unveiled notably poorer levels of nutritional knowledge, with an insufficient level of 37%, satisfactory at 59.3%, and good at 3.7% [87]. It is noteworthy that research indicates an ongoing insufficiency in the nutrition education provided to medical students [88]. Additionally, in a cross-sectional study conducted in 2017, a strong, statistically significant inverse correlation was identified between BMI and the percentage of correct responses pertaining to nutritional knowledge [89]. Consequently, it is discerned that a noteworthy proportion, constituting one-quarter of participants exhibiting abnormal BMI, may have exerted an influence on the inadequacy observed in nutritional knowledge.

A distinctive observation in our findings was the notable increase, as the academic year progressed, in the percentage of students categorized as having insufficient nutritional knowledge—a result warranting scrutiny. One plausible interpretation is connected with the fact that there is simply an increased number of participants in the second period—more individuals completed the survey, therefore individuals who are academically less proficient and initially less inclined to participate may have refrained from involvement in the first assessment, indicating a characteristic lack of interest. Consequently, it is conceivable that this subgroup, displaying an initial hesitancy to engage, may exhibit lower academic performance due to their disinterest and, consequently, possess insufficient nutritional knowledge, which presented itself during the survey at the end of the year. 

However, this trend may be a reflection of the escalation of academic demands, particularly evident during examination periods at the end of the academic year, which poses a notable challenge to the dietary habits of first-year medical students. A study conducted in 2019 supports this trend, indicating that, during the examination period, there was a decline in diet quality [90]. This decline was evidenced by a lower general diet quality index, reduced intake of fruits and vegetables, increased consumption of fast food, and heightened challenges in maintaining a healthy diet. Moreover, the heightened stress levels associated with rigorous study schedules may foster an inclination towards unhealthy dietary choices [91]. Concurrently, the increased reliance on caffeine for its stimulant effects during prolonged study sessions may compound the situation by contributing to disrupted sleep patterns and irregular eating habits [70]. Caffeine, while temporarily alleviating fatigue, can subsequently lead to increased levels of stress and anxiety [68]. The deleterious effects of stress, compounded by the cumulative impact of academic pressures, may engender unhealthy coping mechanisms, such as binge eating or opting for nutritionally deficient food options for the sake of convenience and emotional regulation [92]. As stress becomes a pervasive element of academic life, its influence on dietary decisions and the adoption of unhealthy coping mechanisms [93] underscores the need for targeted interventions and support mechanisms to mitigate the potential negative impact on the well-being of medical students [94]. Addressing stress management strategies and fostering healthier coping mechanisms can contribute to promoting more resilient and balanced dietary habits amidst the academic challenges faced by these students [95].

### 4.4. Differences between Sleep Quality and Nutritional Knowledge Levels

The primary objective of our study was to explore the correlation between dietary habits and sleep quality among students enrolled in a medical university at the beginning and the end of an academic year. Despite different levels of nutritional knowledge, a uniform observation emerged: all groups exhibited a poor total quality of sleep. However, a distinctive trend materialized by the end of the academic year, wherein students characterized by insufficient nutritional knowledge manifested the most compromised sleep quality, while those with good nutritional knowledge demonstrated a notably superior quality of sleep in comparison to their counterparts. Additionally, statistically significant distinctions were noted, elucidating that students possessing insufficient nutritional knowledge reported the highest level of sleep disturbance, exhibited the lowest habitual sleep efficiency, and presented an elevated frequency in the utilization of sleep medication.

Our findings are consistent with previous research on the general population. A 2021 systematic review reported an association between the consumption of healthy foods and improved sleep quality, while a higher intake of processed and free-sugar rich foods was linked to poorer sleep features [96]. Furthermore, a study conducted in 2022 on Spanish university students unveiled significant findings, identifying that possessing a dietary regimen necessitating modification, particularly one characterized as unhealthy, emerged as a predictive factor for diminished sleep quality [97].

The discernible decline in sleep quality among students with insufficient nutritional knowledge by the culmination of the academic year beckons an investigation into the possible interconnections between dietary habits and compromised sleep. One plausible explanation may be that inadequate nutritional knowledge may lead to less optimal dietary choices, potentially impacting sleep patterns [98]. A deficiency in understanding the nutritional requirements for fostering good sleep hygiene could result in suboptimal food selections that influence aspects such as melatonin production [99] or the regulation of sleep-inducing neurotransmitters [33]. Moreover, certain dietary components, such as the consumption of caffeine or stimulants close to bedtime, may contribute to increased restlessness during sleep, impacting the overall continuity and quality of rest [100]. The lower habitual sleep efficiency among the group with insufficient nutritional knowledge suggests that dietary habits could indeed play a role in the effectiveness of sleep. Certain food choices and their timing may influence the body’s ability to transition through sleep cycles smoothly [101]. For instance, the consumption of heavy or rich foods close to bedtime may lead to indigestion and discomfort, potentially disrupting the normal sleep architecture and resulting in reduced sleep efficiency. Additionally, it is probable that individuals with suboptimal dietary habits may experience challenges in naturally regulating sleep, necessitating the reliance on sleep medications to address persistent sleep difficulties [102]. The connection between dietary choices, sleep disturbances, and the subsequent need for sleep medications underscores the intricate relationship between nutritional knowledge, dietary behaviors, and the multifaceted nature of sleep outcomes.

Our investigation revealed a noteworthy escalation in the utilization of sleep-inducing medications, with a statistically significant difference observed. Notably, as the academic year concluded, there was a substantial increase in the requirement for sleep medications. This trend was particularly pronounced in cohorts characterized by suboptimal nutritional knowledge, both at the beginning and the end of the academic year. In 2019, a study identified a robust and statistically significant correlation between favorable dietary habits and reduced utilization of sleep medication [103]. This phenomenon may be attributed to the propensity of individuals possessing enhanced nutritional knowledge and healthier dietary habits to exhibit a reduced reliance on sleep medication and manifest superior stress coping mechanisms [104]. Such individuals ostensibly maintain a heightened standard of sleep hygiene, contributing to their diminished reliance on sleep medications. It is noteworthy that, in any studied group, the utilization of sleep medication remained higher than in the aforementioned general population studies: in Germany the score was 0.14 and in China it was 0.11, while in our study it progressed from 0.30 to 0.54 by the end of the academic year [64,65].

Furthermore, a comprehensive nationwide study conducted in Japan in 2019 demonstrated a higher prevalence of sleep disturbances associated with less healthy dietary behaviors [105]. This finding aligns with our study’s results, wherein individuals with insufficient nutritional knowledge exhibited more pronounced sleep disturbances, with the disparities between knowledge groups becoming more evident by the conclusion of the academic year. Additionally, the findings of another 2019 study indicate that less than 10% of students manage to achieve 8 h of sleep before final exams [106]. This aligns with our study’s results, where the observed decline in sleep duration towards the end of the academic year corresponds with the heightened stress levels typically experienced during this period. It is noteworthy that respondents characterized by insufficient nutritional knowledge did not demonstrate significant changes, implying a persistently suboptimal sleep duration throughout the year.

Conversely, the substantial enhancement in sleep quality observed among students with good nutritional knowledge implies that well-informed dietary practices may positively contribute to overall sleep health. A diet rich in sleep-promoting nutrients, such as tryptophan [107], magnesium [108], and complex carbohydrates [109], could potentially influence the regulation of sleep–wake cycles and improve the overall quality of sleep. Additionally, informed dietary choices may mitigate factors contributing to sleep disturbances, such as excessive caffeine intake or the consumption of stimulating foods close to bedtime. Nevertheless, it is salient to observe that possessing proficient nutritional knowledge did not suffice for medical students to attain a good quality of sleep, which is an alarming outcome warranting further investigation.

### 4.5. Other Possibly Influential Factors

The investigation into the dynamics influencing the sleep quality and nutritional knowledge of first-year medical students necessitates a comprehensive consideration of various determinants within the academic, social, and psychological domains. The onerous demands inherent in the academic curriculum, coupled with the rigorous workload, impart a significant stressor upon these students, potentially manifesting in disrupted sleep patterns [110]. Additionally, the pervasive influence of social pressures, encompassing the imperative to adhere to academic expectations and establish interpersonal connections, may contribute to a heightened psychological strain [111]. The amalgamation of these stressors may engender compromised mental health, thereby affecting both the quality of sleep and nutritional habits [112]. Culturally embedded expectations [113,114], individual coping mechanisms [115,116,117], and familial influences [118] represent a subset of variables that may exert substantial influence on the holistic well-being of these students. It is essential to acknowledge that, due to the nature of the statistical analysis employed, the influence of confounding variables was minimized. However, it is plausible that some factors not considered in this study may still correlate with nutritional knowledge and independently affect sleep, contributing to the multifaceted nature of the challenges faced by first-year medical students.

### 4.6. Future Implications

The outcomes of this study underscore the critical need for policy interventions to enhance the well-being of medical students, with a focus on the interplay between dietary habits and sleep quality. Policymakers should consider allocating funding for the development and implementation of educational programs within medical curricula that integrate comprehensive knowledge about nutrition, mental health, and stress management. These programs could empower students to make healthier dietary choices and equip them with effective stress-coping mechanisms. Additionally, establishing dedicated wellness faculties or support services within medical universities could provide holistic support to students, addressing not only nutritional education but also mental health and work–life balance. Furthermore, there is a clear need for further research exploring specific dietary factors influencing sleep quality, allowing for evidence-based guidelines tailored to the unique challenges faced by medical students. Longitudinal studies could provide insights into the dynamic nature of these associations over the course of medical education. A collaborative effort of policymakers and educational institutions may help foster a supportive environment that promotes the overall well-being of medical students.

### 4.7. Limitations of the Study

While this study offers valuable insights, it is imperative to acknowledge its inherent limitations. The research cohort, comprising students solely from Wroclaw Medical University, represents a specific subset of the population, limiting the generalizability of the findings to a broader societal context. Another constraint stems from the online survey methodology, introducing the possibility of participant misinterpretations of questions, despite efforts by researchers to provide clear instructions. Consequently, the behavioral characteristics under scrutiny should not be extrapolated to the wider adult population. Furthermore, despite the longitudinal nature of the study, there is a slight variation in the study sample between the two examined periods. Consequently, the precise comparison of participants in both periods through a dependent test is unattainable, due to the complete anonymization of data collection. A more comprehensive insight into lifestyle dynamics and their implications could be attained through a longitudinal study design with partial anonymization, enabling the use of dependent sample statistics. The study’s scope, limited to nutritional knowledge and sleep quality, may constrain the comprehensive understanding of determinants, as significant factors including academic stress, social pressures, and overall mental health remain unexplored, thereby potentially limiting the interpretation and applicability of the findings. It is recommended that future research endeavors encompass a more diverse and extensive participant pool to augment the applicability and depth of the findings presented in this paper.

## 5. Conclusions

This study investigated the link between nutritional knowledge and sleep quality in first-year students at Wroclaw Medical University at the beginning and the end of the academic year. Analysis of BMI revealed a prevalence of abnormal BMI in around one-quarter of the participants, with favorable outcomes compared to existing research. Despite the recognized importance of a consistent and healthy sleep routine, the results indicated poor sleep quality at both time points, with a notable decline by the end of the academic year. The prevalence of poor sleep quality aligns with global studies on medical students, indicating a concerning trend. Factors contributing to the observed deterioration include rigorous academic demands, final examinations, heightened stress, and potential lifestyle factors like increased caffeine intake and screen time. Our study revealed that over 80% of participants demonstrated sufficient or good nutritional knowledge. Notably, an increase in students with insufficient nutritional knowledge as the academic year progressed may be attributed to heightened academic demands, especially during examination periods, impacting the dietary habits of first-year medical students. Elevated stress levels during intense study schedules may lead to unhealthy dietary choices, amplified by increased caffeine intake. Unhealthy coping mechanisms, like binge eating, may further compound the impact of stress. Addressing stress management strategies and fostering healthier coping mechanisms are crucial to supporting the well-being of medical students facing the challenges of their academic journey. While all groups demonstrated poor sleep quality, students with insufficient nutritional knowledge showed a significant decline in sleep quality by the year’s end. This suggests a potential connection between suboptimal dietary choices and compromised sleep. Conversely, students with good nutritional knowledge exhibited improved sleep quality, emphasizing the positive impact of informed dietary practices; however, they still did not obtain a good quality of sleep.

## Figures and Tables

**Table 1:** Characteristics of study participants.

Variables	Beginning of the AY, *N* = 570 [IQR], (%)	End of the AY, *N* = 705 [IQR], (%)
**Age**	19.7 [19.0–20.0]	20.0 [19.0–20.0]
**Gender**		
Male	130 (22.8)	190 (27.0)
Female	440 (77.2)	515 (73.1)
**Place of residence**		
Rural area	154 (27.0)	145 (20.6)
City <50,000 *	137 (24.0)	146 (20.7)
City 50,000–100,000 *	73 (12.8)	79 (11.2)
City 100,000+ *	206 (36.2)	335 (47.5)
**Body Mass Index**		
Mean	21.7 [19.5–23.4]	21.6 [19.4–23.1]
Underweight	75 (13.2)	79 (11.2)
Normal weight	414 (72.6)	542 (76.9)
Overweight	81 (14.2)	84 (11.9)
**Nutritional knowledge mark**		
Insufficient	49 (8.6)	138 (19.6)
Sufficient	361 (63.3)	385 (54.6)
Good	160 (28.1)	182 (25.8)
**Sleep quality**		
Good	214 (37.5)	244 (34.6)
Poor	356 (62.5)	461 (65.4)

Note: *N* is the number of observations; * Number of inhabitants.

**Table 2:** Overall sleep quality between study intervals.

	Beginning of the AY			End of the AY				
	Mean	Mdn	IQR	Mean	Mdn	IQR	*p*-Value	Z
**TOTAL**	6.86	6.0	4.0–9.0	7.38	7.0	5.0–10.0	**0.0157**	2.424
**DURAT**	0.45	0.0	0.0–1.0	0.79	1.0	0.0–1.0	**<0.0001**	7.169
**DISTB**	1.19	1.0	1.0–2.0	1.22	1.0	1.0–2.0	0.4041	0.956
**LATEN**	2.08	2.0	1.0–3.0	1.86	2.0	1.0–3.0	**0.0063**	2.786
**DAYDYS**	1.45	2.0	0.0–2.0	1.43	2.0	0.0–2.0	0.6801	0.433
**HSE**	0.45	0.0	0.0–1.0	0.53	0.0	0.0–1.0	0.5998	0.647
**SLPQUAL**	0.94	1.0	1.0–1.0	1.01	1.0	1.0–1.0	0.1089	1.831
**MEDS**	0.30	0.0	0.0–0.0	0.54	0.0	0.0–1.0	**<0.0001**	5.842

Note: TOTAL—total score, DURAT—sleep duration, DISTB—sleep disturbance, LATEN—sleep latency, DAYDYS—daytime dysfunction, HSE—habitual sleep efficiency, SLPQUAL—sleep quality, MEDS—use of sleeping medication. Significant *p*-values are presented in bold.

**Table 3:** Differences between sleep quality and nutritional knowledge levels.

	Beginning of the AY						End of the AY						Beginning vs. End	
	Mean	Mdn	IQR	*p*-Value	H	Post hoc	Mean	Mdn	IQR	*p*-Value	H	Post hoc	*p*-Value	Z
**TOTAL**														
Insufficient	7.82	8.0	5.0–10.0				8.41	8.0	5.0–11.0				0.3026	−1.029
Sufficient	6.72	6.0	4.0–9.0	0.1105	4.405	N/S	7.43	7.0	5.0–10.0	**0.0001**	19.732	A > B > C	**0.0127**	−2.491
Good	6.89	6.0	4.0–9.0				6.49	6.0	4.0–9.0				0.3885	0.862
**DURAT**														
Insufficient	0.65	0.0	0.0–1.0				0.80	1.0	0.0–1.0				0.3112	−1.011
Sufficient	0.46	0.0	0.0–1.0	0.1482	3.818	N/S	0.81	1.0	0.0–1.0	0.7988	0.449	N/S	**<0.0001**	−5.834
Good	0.39	0.0	0.0–1.0				0.75	0.0	0.0–1.0				**0.0001**	−3.878
**DISTB**														
Insufficient	1.33	1.0	1.0–2.0				1.51	2.0	1.0–2.0				0.0997	−1.645
Sufficient	1.20	1.0	1.0–2.0	**0.0261**	7.295	N/S	1.19	1.0	1.0–2.0	**<0.0001**	40.276	A > B, C	0.9566	0.054
Good	1.12	1.0	1.0–1.0				1.05	1.0	1.0–1.0				0.2314	1.196
**LATEN**														
Insufficient	2.06	2.0	1.0–3.0				2.00	2.00	1.0–3.0				0.7128	0.367
Sufficient	2.06	2.0	1.0–3.0	0.8013	0.443	N/S	1.92	2.0	1.0–3.0	0.0876	4.870	N/S	0.1596	1.406
Good	2.13	2.0	1.0–3.0				1.64	1.0	0.0–3.0				**0.0015**	3.167
**DAYDYS**														
Insufficient	1.49	2.0	1.0–2.0				1.30	1.0	0.0–2.0				0.2683	1.105
Sufficient	1.42	2.0	0.0–2.0	0.5655	1.140	N/S	1.49	2.0	0.0–2.0	0.1724	3.516	N/S	0.3973	−0.901
Good	1.53	2.0	1.0–2.0				1.39	2.0	0.0–2.0				0.2371	1.182
**HSE**														
Insufficient	0.61	0.0	0.0–1.0				0.78	0.0	0.0–1.0				0.3564	−0.921
Sufficient	0.44	0.0	0.0–1.0	0.6181	0.962	N/S	0.53	0.0	0.0–1.0	**0.0001**	19.691	A > C	0.2785	−1.084
Good	0.44	0.0	0.0–1.0				0.32	0.0	0.0–0.0				**0.0289**	2.184
**SLPQUAL**														
Insufficient	1.06	1.0	1.0–1.0				1.04	1.0	1.0–2.0				0.8932	0.133
Sufficient	0.92	1.0	1.0–1.0	0.4918	1.420	N/S	1.01	1.0	1.0–1.0	0.9139	0.180	N/S	0.1289	−1.518
Good	0.94	1.0	1.0–1.0				1.00	1.0	1.0–1.0				0.2952	−1.046
**MEDS**														
Insufficient	0.61	0.0	0.0–1.0				0.97	1.0	0.0–2.0				**0.0229**	−2.274
Sufficient	0.23	0.0	0.0–0.0	**0.0006**	14.830	A > B	0.49	0.0	0.0–1.0	**<0.0001**	50.293	A > B, C	**<0.0001**	−4.930
Good	0.35	0.0	0.0–0.0				0.33	0.0	0.0–0.0				0.9586	−0.051

Note: TOTAL—total score, DURAT—sleep duration, DISTB—sleep disturbance, LATEN—sleep latency, DAYDYS—daytime dysfunction, HSE—habitual sleep efficiency, SLPQUAL—sleep quality, MEDS—use of sleeping medication; A—insufficient, B—sufficient, C—good; N/S—not significant. Significant *p*-values are presented in bold.

## Data Availability

The datasets used and analyzed during the current study are available from the corresponding author upon reasonable request. The data are not publicly available due to to the inclusion of information that could compromise the privacy of research participants.
